# Mechanisms of Immune Evasion in Multiple Myeloma: Open Questions and Therapeutic Opportunities

**DOI:** 10.3390/cancers13133213

**Published:** 2021-06-28

**Authors:** Cirino Botta, Francesco Mendicino, Enrica Antonia Martino, Ernesto Vigna, Domenica Ronchetti, Pierpaolo Correale, Fortunato Morabito, Antonino Neri, Massimo Gentile

**Affiliations:** 1 Hematology Unit, Annunziata Hospital of Cosenza, 87100 Cosenza, Italy; f.mendicino@aocs.it (F.M.); enricaantoniamartino@libero.it (E.A.M.); ernesto.vigna@aocs.it (E.V.); 2 Unit of Hematology, Department of Health Promotion, Maternal-Infant, Internal and Specialized Medicine of Excellence G. D’Alessandro, University of Palermo, 90127 Palermo, Italy; 3 Department of Oncology and Hemato-Oncology, University of Milan, 20122 Milan, Italy; domenica.ronchetti@unimi.it (D.R.); antonino.neri@unimi.it (A.N.); 4 Medical Oncology Unit, Grand Metropolitan Hospital “Bianchi-Melacrino-Morelli”, 89124 Reggio Calabria, Italy; pierpaolo.correale@ospedalerc.it; 5 Hematology and Bone Marrow Transplant Unit, Hemato-Oncology Department, Augusta Victoria Hospital, East Jerusalem 91191, Israel; fmorabito@avh.org; 6 Biothecnology Research Unit, AO of Cosenza, 87100 Cosenza, Italy; 7 Hematology, Fondazione IRCCS Cà Granda, Ospedale Maggiore Policlinico, 20122 Milano, Italy

**Keywords:** multiple myeloma, tumor immunology, anti-cancer immune response, immunotherapy, monoclonal antibodies

## Abstract

**Simple Summary:**

The growing interest in immunotherapy for the treatment of multiple myeloma demands a deep knowledge of the complex interactions between malignant and immune cells within the bone marrow. Indeed, understanding the cellular and molecular mechanisms underlying this network should represent the basis for the design of novel patient-oriented biological therapeutic approaches. Here, we describe the role of the main immune components of the myeloma niche along disease evolution and their implication in impairing/improving the response to anti-cancer treatments. Additionally, we provided an overview of the potential weakness of this pro-tumor interplay, evidencing novel therapeutic opportunities, which deserve future clinical investigations.

**Abstract:**

Multiple myeloma (MM) is the second most common hematologic malignancy, characterized by a multi-step evolutionary path, which starts with an early asymptomatic stage, defined as monoclonal gammopathy of undetermined significance (MGUS) evolving to overt disease in 1% of cases per year, often through an intermediate phase known as “smoldering” MM (sMM). Interestingly, while many genomic alterations (translocation, deletions, mutations) are usually found at early stages, they are not sufficient (alone) to determine disease evolution. The latter, indeed, relies on significant “epigenetic” alterations of different normal cell populations within the bone marrow (BM) niche, including the “evasion” from immune-system control. Additionally, MM cells could “educate” the BM immune microenvironment (BM-IM) towards a pro-inflammatory and immunosuppressive phenotype, which ultimately leads to disease evolution, drug resistance, and patients’ worse outcome. Indeed, it is not a case that the most important drugs for the treatment of MM include immunomodulatory agents (thalidomide, lenalidomide, and pomalidomide) and monoclonal antibodies (daratumumab, isatuximab, and elotuzumab). On these bases, in this review, we describe the most recent advances in the comprehension of the role of the different cells composing the BM-IM, and we discuss the potential molecular targets, which could represent new opportunities to improve current treatment strategies for MM patients.

## 1. Introduction

In recent years, the increased knowledge in cancer immunotherapy has focused many research efforts on the interaction between cancer cells and the immune microenvironment (IM), supporting the concept that IM plays a crucial role in tumor evolution and progression [1,2,3]. Different preclinical studies, in both solid and hematologic oncology, demonstrated the capability of the immune system to maintain tumors for a long time in a state of equilibrium, where clonal expansions are antagonized by adaptive immunity (equilibrium phase of immunoediting) [4,5]. These concepts support the hypothesis that patients with very early stage or undetectable cancers may have tumor cells, which, while owing all the genomic alterations typical of advanced diseases, (potentially) never give rise to an active/aggressive disease (such as clonal hematopoiesis of indeterminate potential) [6]. Multiple myeloma (MM), the second most common hematologic malignancy, presents a readily detectable premalignant stage, thus representing a unique setting to study this phenomenon. Indeed, MM is a multi-step evolutionary disease characterized by an early stage defined as monoclonal gammopathy of undetermined significance (MGUS), which evolves into the overt disease often by an intermediate asymptomatic phase or “smoldering” MM (sMM) [7,8,9]. While malignant plasma cells (PCs) usually present key genomic features of active MM (including chromosomal aberrations such as translocations involving IgH or hyperdiploidy/*TP53* mutations/MYC translocations) [9], only 1% MGUS and 10% sMM per year eventually evolve to overt MM, supporting the idea that “cell-intrinsic” factors (i.e., genomic alterations) are not sufficient, alone, for disease evolution. The latter, indeed, also relies on significant epigenetic alterations of different “normal” cell populations within the bone marrow (BM) niche. Accordingly, the most active drugs for MM treatment or for reducing the risk of evolution from sMM to MM include agents with strong activity on the microenvironment, such as immunomodulatory drugs (IMiDs) or monoclonal antibodies [10,11]. In this context, much evidence supports the critical role played by stromal cells [12,13] and different cells of the IM (such as dendritic cells [14], myeloid-derived suppressor cells (MDSCs) [15,16], natural killer [17], and specific lymphocytes subclasses including the pro-inflammatory Th17 cells [18,19]) as well as BM-extrinsic drivers such as the microbiota [20] in driving MM evolution. Gut microbiota, particularly by promoting chronic inflammation and/or immune suppression, could drive non-aerodigestive tract malignancies with mechanisms that are still under active investigation and have been recently identified as a new player in determining tumor progression [20,21]. Along this line, several studies already demonstrated a dysbiosis in MM patients as compared to MGUS or healthy donors, while an increase in the abundance of Prevotella species in the gut of sMM mice quickly progressing to MM, with a mechanism dependent on Th17 activation, has been recently observed [20]. The cross-talk between MM cells and immune cells (ICs) generates an inflammatory/immunosuppressive milieu, which provides a significant survival advantage for MM cells in terms of both resistance to conventional chemotherapy and immunotherapeutic failure [22,23,24]. Indeed, despite the recent introduction of novel drugs, which improved the clinical outcome of MM patients, the disease remains incurable, and novel “microenvironment oriented” strategies are eagerly awaited [11]. Accordingly, the possibility to modulate the inflammatory microenvironment as well as the immune system by either recovering the tumor-associated immunosuppression or enhancing the anti-cancer immune-response represents an emerging and effective opportunity to induce long-lasting clinical benefits in different malignancies [25].

Within this work, we describe the most recent advances in comprehending the role of the different cells composing the myeloma-associated IM, with a specific focus on the molecular and cellular mechanisms involved in immune evasion. Next, we discuss targets with potential translational relevance for the development of new immunotherapeutic strategies for the treatment of MM patients.

## 2. Myeloid Compartment

### 2.1. MDSCs

MDSCs are usually described as a mixture of cells in different stages of myeloid differentiation able to impair innate and adaptive immune responses through several mechanisms, including L-arginine depletion, oxidative stress, CD8^+^ T-cell apoptosis, and imbalance of Th1/Th17/Treg response [15,22]. These cells are commonly defined as a CD11b^+^CD33^+^HLADR^-/lo^ subset within density gradient separated mononucleated cells, which expand in patients with cancer. MM cells could induce MDSC development, whereas, in turn, MDSCs foster tumor growth and chemo-resistance and induce immune evasion [15].

An increase in MDSCs in peripheral blood and BM aspirates from MM patients (as compared to MGUS or healthy donors) has been reported [26,27]. Furthermore, along with disease evolution, an increase in the activation of the JAK/STAT signaling in response to MM cells exposure has been observed, which, coupled with MDSCs’ capability to differentiate into “osteoclast-like” cells to deplete arginine from the microenvironment through overexpression of arginase-1 and to increase nitric oxide production, supports the establishment of a proinflammatory and tolerogenic niche as well as the generation of lytic bone lesions [15,24,25,28]. 

Therefore, it is clear that MDSC should be considered the main target for anti-MM therapy. However, contrasting data report the capability of several anti-MM drugs to potentiate (dexamethasone, melphalan, and cyclophosphamide) or to deplete (daratumumab) MDSCs within the BM microenvironment. This point is of the utmost relevance, especially if we take into account the plethora of immunotherapies currently available for MM, including monoclonal antibodies, CAR T cells, and T cell engager bispecific antibodies, making mandatory the identification of an optimal strategy for immunomonitoring. We and others recently demonstrated that in MM patients, mature neutrophils should be considered the true MDSCs [16,29,30,31]. Indeed, by impairing the final part of the differentiation through a strong epigenetic remodeling, MM cells educate differentiating neutrophils to exhibit a pro-inflammatory and pro-tumoral transcriptomic and cytokine profile able to impair lymphocytes response and bispecific drugs activity. We demonstrated that this process could be reverted by using hypomethylating agents, making this class of agents be taken into account for combinatory strategy purpose.

Along this line, neutrophil to lymphocyte ratio (NLR) at diagnosis can predict the outcome in newly diagnosed and post-ASCT MM patients [16,32,33].

**Figure 1:**
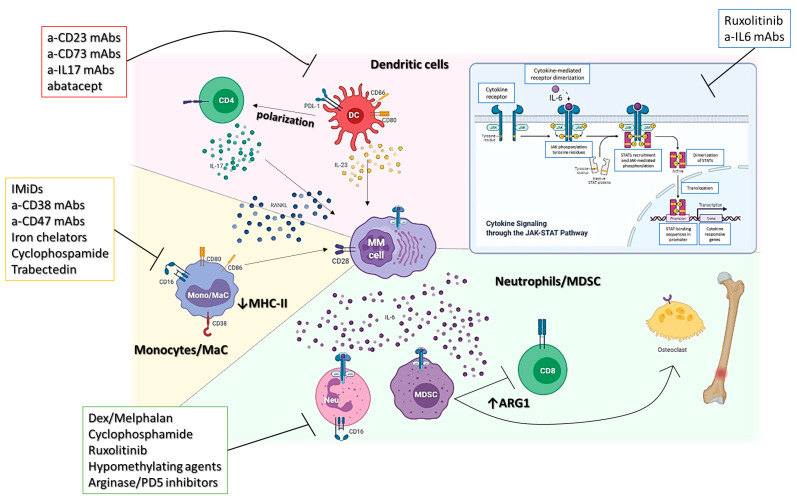
Role of myeloid cells in MM niche. Cartoon representing the main relationships among immune cells of myeloid origin and MM cells. Therapeutic opportunities are reported according to the pathways/cells they interact with.

Overall, these data support the role of an MDSC-oriented therapeutic strategy, which could include JAK/STAT (ruxolitinib), arginase, or phosphodiesterase-5 inhibitors, as well as hypomethylating agents to relieve the immunosuppression and support the activity of concomitant anti-MM (immuno)therapies (Figure 1).

### 2.2. Monocytes/Macrophages

Monocytes and macrophages are among the most important regulators of cancer-associated inflammation. Their role in cancer progression has been widely described in solid tumors and, more recently, in hematological malignancies, including MM. Indeed, it has been suggested that within the BM microenvironment, tumor-associated monocytes and macrophages (TAMs) could protect MM cells from therapy-induced apoptosis and promote neo-angiogenesis and immune-escape [22,23]. Additionally, several reports confirmed TAMs’ role in inducing resistance to commonly used anti-MM drugs such as melphalan or bortezomib [34,35].

Currently, a plethora of surface markers have been used to describe TAMs (including CD14, CD206, CD68, and CD163), which are used to identify at least two types of functional macrophage states: M1 (inflammatory or “classically activated”), activated during infections and M2 (suppressive, “alternative pathway”) involved in angiogenesis and wound healing. Of note, M1 and M2 should be considered the two extremes of a continuum; TAMs indeed often present a mixed transcriptional profile [36,37,38]. Interestingly, monocytes show a similar polarization pattern, which relies on the expression of CD14 and CD16; in particular, we recognize: classical (CD14^+^CD16^−^), intermediate (CD14^+/−^ CD16^low^), and non-classical monocytes (CD14^−^CD16^+^), the latter being considered a tumor-promoting phenotype [39]. Due to the lack of shared detection methods, the percentage of TAMs within MM patients’ BM has been reported to be highly variable (from near 0 up to 25%), increasing during evolution from MGUS to MM, with reports indicating a worse prognosis for patients with a high CD163^+^ and CD206^+^ TAM infiltration [40,41,42]. A recent single-cell study revealed that even if reduced in MGUS compared to most advanced stages, mature monocytes/macrophages are already dysfunctional, presenting a phenotypic shift leading to the loss of MHC type II surface representation impairing their antigen-presenting cell capability [43].

On these premises, several macrophages depleting/reprogramming therapies are under active investigation. The IKZF1-IRF4/IRF5 axis is necessary to drive the pro-tumoral polarization of macrophages, and its targeting exerted by IMiDs demonstrated the recovery of an anti-tumor functional status [44]. This event could contribute to the brilliant clinical results achieved by the combination of IMiDs with myeloma targeting monoclonal antibodies such as daratumumab and isatuximab, whose activity relies in part on the presence of functional macrophages [11,45,46]. Further drugs, such as anti-CD47 antibodies [47], iron chelators [48], cyclophosphamides [46], and trabectedin [49], are currently under investigation for their activity on MM-TAMs (Figure 1).

### 2.3. Dendritic Cells

Dendritic cells (DC) are key antigen-presenting cells (APCs), which work as a bridge between innate and adaptive immunity. They are commonly divided into two subgroups according to their function: myeloid DCs (mDCs) and plasmacytoid DCs (pDCs). pDCs are specialized in the production of type I IFN in response to specific stimuli such as viruses; on the other hand, by releasing particular context-dependent cytokines and through MHC-dependent and independent cell-to-cell interaction, mDCs orchestrate the differentiation and polarization of different populations of T lymphocytes, such as Th1, Th2, Th17, CTL, and Tregs [14,22,23,28,50]. Additionally, mDCs can also interact with NK and B cells and are involved in the development of local and systemic inflammation as well as autoimmune disease and cancer [14,22,23,28,50]. It is essential to figure out how DCs are usually regulated and how this regulation may be compromised within cancer IM to understand their potential dual role (as tumor-suppressors or tumor-promoting cells) in MM fully. Indeed, DCs extensively infiltrate (up to 10%, accumulating during MGUS to MM evolution [23]) the BM of MM patients, differentiating from local progenitors or monocytes attracted by the inflammatory BM microenvironment. Within the BM, through different epigenetic modifications [14], DCs are reprogrammed to (1) directly support MM cells proliferation by producing growth cytokines including IL-6, RANK-L, and APRIL with a mechanism dependent on the activation of proinflammatory pathways (p38 and NFkB among others); (2) favor the osteoclastogenesis process (being themselves able to differentiate into osteoclast-like cells); (3) induce Th17 polarization and, mainly, the secretion of IL-17A, which works both as a direct growth factor for MM and as a proinflammatory/bone lysis inducer cytokine; (4) promote neoangiogenesis; (5) genomic instability; finally, (6) protect MM cells from drug-induced (melphalan and bortezomib) apoptosis through the activation of the CD28/CD86 axis [14,19,22,23,51,52]. In this context, several DCs subsets express PD-L1 and the immunosuppressive enzyme indoleamine-2,3-dioxygenase (IDO), which contribute in attenuating a possible tumor specific CTL response within the BM-IM [23,53]. Therefore, it is clear that targeting the DC-MM crosstalk could be of the utmost importance to recover the physiological antigen presentation functionality fully. Indeed, several transcriptomic studies suggest that either CD73 (on pDCs) or IL23 (on mDCs) might be involved in the establishment of an immunosuppressive/proinflammatory BMM, and that their targeting could rescue the immunological competence of DCs [14,23,51]. Additionally, the functional inhibition of the CD28/CD80/CD86 axis with CTLA4Ig as well as the use of anti-IL17 mAbs are currently being explored clinically for the treatment of MM patients (Figure 1) [19,54,55].

## 3. Lymphoid Compartment

### 3.1. T Helper Response

Naïve T helper cells polarize, depending on the microenvironment in which they live, into fully effector cells, which mainly belong to the Th1, Th2, Th17, and regulatory T cells subsets. Th1 cells promote a cytotoxic phenotype and are involved in the defense against virus, intracellular bacteria as well as cancer cells, while Th2 lymphocytes mainly promote a humoral response and also orchestrate the immune response against parasites through the activation of cells of the innate immune response (mast cells and eosinophils) and by inducing IgE class-switch in B cells. In this context, IFN-γ/TGF-β and IL-3/4/5 are the main markers of Th1 and Th2 response, respectively. 

Overall, it has been reported that a higher percentage of CD4+ T cells can be found in the BM of patients with newly diagnosed MM or high-risk SMM (but not with MGUS) as compared to HD [28].

Few (and contrasting) data have been reported on the role of the Th1/Th2 response in MM with no evident alteration in the distribution of those populations in patients as compared to healthy subjects. Interestingly, a reduced Th1/Th2 ratio is associated with worse ISS stage, LDH, and serum β2-microglobulin. However, and interestingly, an increase in Th2 response has been observed after lenalidomide and pomalidomide treatment. Specifically, patients who developed an IMiDs-related skin rash obtained the best results in the anti-cancer response, raising the attention again on the importance of a Th2 response against MM [56].

More recently, the balance between Th17 and Tregs in the BM microenvironment of patients with MM has gained a renewed interest. Physiologically, Th17 cells are involved in the generation of chronic inflammation, could activate neutrophils, and trigger the defense machinery against extracellular bacteria and fungi by producing, among others, IL-17, IL6, IL-22, and TNF-α [18,22,24]. On the other hand, Tregs are involved in self-tolerance induction and maintenance, autoimmunity prevention, and repression of immune response to avoid damages from an unwanted/overwhelmed inflammatory response through cell-to-cell contact and secretion of IL-10 and TGF-beta [22,24]. 

**Figure 2:**
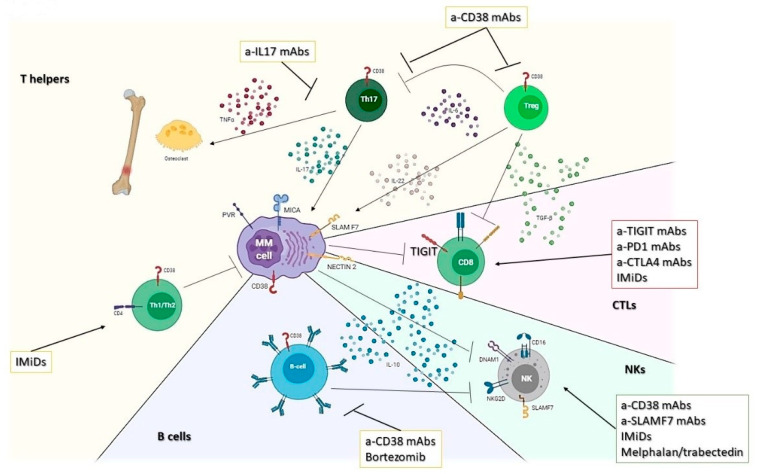
Role of B, T, and NK lymphocytes in MM niche. Cartoon representing the interplay existing between bone-marrow-infiltrating lymphocytes and MM cells. Therapeutic opportunities are reported according to the pathways/cells they interact with.

Multiple experimental evidence suggests that IL-17 (and Th17) plays a pivotal role in MM development and progression. Indeed, several authors recently reported on the capability of IL-17, induced by several changes in gut microbiota, to promote disease evolution from SMM to MM [20]. Additionally, Th17 could promote cell proliferation and migration, neoangiogenesis, immune evasion, and bone disease in MM [14,18,19]. The role of Tregs instead is still controversial: there are few reports describing the amount of Tregs infiltration in MM patients (no differences with BM from healthy donors), and no precise data are currently available on their role on patients’ outcome or response to treatment [57,58]. Recently, a single cell study on BM microenvironment among MM evolution identified an increase in Treg lymphocytes compared to HD in all the different phases of MM evolution (starting from MGUS), suggesting that T cell dysfunction is an early event [43]. Of note, Tregs usually express high levels of CD38, a target of isatuximab and daratumumab, thus potentially increasing the T cell anti-MM response induced by these agents (Figure 2) [59].

### 3.2. Cytotoxic T Lymphocytes (CTLs)

Cytotoxic T lymphocytes (CD8^+^ T cells) are considered the effectors of the adaptive immune system, being in charge of the protection against intracellular infections (either bacteria or virus) and the elimination of malignant cells. Interestingly, once the target cells have been eliminated, few antigen-specific CTLs survive transformed in memory cells, ready to be re-activated if and when needed [22,23,25,28]. In MM patients, CTLs have been found to be increased in both MGUS and symptomatic MM (with respect to healthy donors) but dysfunctional (in term of proliferation and cytotoxic activity), presenting a reduced capability to respond to antigenic stimuli. Indeed, it has been found that MM-specific CTLs are unable to kill MM cells mainly due to several mechanisms, including T cell exhaustion/senescence and the protective effect exerted by the myeloid compartment, which is highly represented within the tumor microenvironment [16,25,60]. The latter mechanism, in particular, partially relies on the induction of CD28 signaling in MM cells by DCs, which reduces the capability of MM cells to present antigens through class-I HLA, thus evading CTL recognition through T cell receptor engagement. On the other hand, different soluble factors secreted by MM or ancillary cells (myeloid/mesenchymal cells) have been found to modulate and eventually abrogate the antitumor activity of CTLs, including TGF-β, IL-10 as well as the immunosuppressive nucleoside adenosine and ADP (dependent on CD38, CD39, and CD73 enzymatic activity) [25,61].

MM-associated T-cells could present a mixed phenotype, which ranges from a “senescent” effector phenotype, characterized by positivity to KLRG-1, CD57, CD160 associated to low or negative CD28, CTLA4, and PD1, up to an “exhaustion” phenotype, which includes the positivity for PD1, CTLA-4, CD57 and the lack of CD28 [22,25,43,60]. Unfortunately, the failure of trials including anti-PD1 mAbs (even if some interesting results have been observed from IMiDs-anti-PD1 combinatory strategies) revealed a dismal clinical role for the axis PDL1-PD1 in MM [61,62,63]. Several trials are now exploring the activity of anti-CTLA4 mAbs alone or in combination with anti-PD1. At the same time, the targeting of the recently recognized immune checkpoint T-cell immunoglobulin and immunoreceptor tyrosine-based inhibition motif (ITIM) domains (TIGIT) seems to play a promising role in the future immunotherapy of MM to provide long-term immunological control of the disease (Figure 2) [64,65].

### 3.3. NK

NK cells are granular CD56^+^CD3^−^ lymphocytes, which represents an essential subset of the innate immune response. They play a key role in the immunity against viral infection, in immune surveillance of cancer, and as effectors of anti-tumor therapies. On the NK surface, we can find activating receptors (such as NKG2D, CD16, 2B4, NKp80, DNAM-1, and natural cytotoxicity receptors) and inhibitory receptors (KIRs, CD94/NKG2A, and ILT2/LIR1/CD85j), which recognition by specific ligands could determine an imbalance between the different intracellular signaling able to activate or inhibit NK activation [25,28,49,66]. KIRs, in particular, by recognizing classical major histocompatibility (MHC), class I molecules efficiently block NK cell activation against MHC-I-expressing normal cells. Once activated, NKs could kill the target cell in several ways: through secretion of cytotoxic granules such as perforin or granzyme B or via death receptors such as Fas and TRAIL-related pathways. Preclinical and clinical findings have demonstrated a central role for NK cells in mediating anti-myeloma activity. Indeed, a decline in NK cell surveillance and cytotoxicity against MM has been observed along with disease evolution from MGUS to later stages [22,43]. Along this line, the NKG2D ligand MICA was found to be reduced in advanced disease compared to MGUS (shedding), while the expression of inhibitory ligands such as class I MHCs (ligands for inhibitory KIR), negligible in early MM, increases in advanced stages. Accordingly, several drugs, including melphalan, doxorubicin, and trabectedin, have been found to increase the expression of activating ligands on MM cells and, consequently, to induce NK-mediated cytotoxicity [49,67,68,69].

A graft vs. myeloma effect has been shown by the differences in post-allogeneic stem cell transplant relapse rates based on the inherited repertoire of KIR genes expressed by donor NK cells, indicating a role for NK cell-mediated suppression of relapse [22,24]. Lastly, NK cells mediate ADCC against myeloma cells in vitro and in vivo, thus enhancing the anti-MM activity of current therapies, which includes mAbs such as the anti-CD38 daratumumab and isatuximab [45] and the anti-SLAMF7 elotuzumab (Figure 2) [70,71]. 

### 3.4. B Lymphocytes

B cells are the main components of humoral immunity and are responsible for the production and secretion of antibodies. B lymphocytes are strongly impaired in MM patients, where a progressive reduction in CD19^+^ cells is accompanied by an impaired functionality with reduced production of polyclonal antibodies and class-switch. Additionally, an increase in regulatory B cells (Bregs) along myeloma evolution from MGUS has been reported, which determine increased release of IL-10 in the microenvironment and inhibition of NK-mediated ADCC [25,28,72,73]. 

Interestingly, Bregs accumulation depends on disease burden, and different approaches to overcome their immunosuppressive effects should be taken into consideration when designing therapeutic combinations. Indeed, they could be targeted by daratumumab (due to their CD19^+^CD24 ^bright^CD38 ^bright^ phenotype) [74] or depleted by bortezomib use (Figure 2) [28,75]. Despite their potential role as immune-suppressors, Bregs are still poorly studied in MM.

## 4. Mesenchymal Stem Cells

Mesenchymal stem cells (MSC) are among the most important constituents of the bone marrow (BM) non-hematopoietic microenvironment and their role as “drivers” of myeloma pathobiology has been largely established [24,76]. While MSC-derived cytokines (including interleukin-6) are fundamental for supporting MM growth, their role in MM evolution still remains to be fully elucidated. Interestingly, MSCs play a fundamental role in the development of MM-associated bone disease [12,13], and at least in vitro, their post-transcriptional regulation through RNA interference could reduce the secretion of RANK ligand, thus impairing the capability of osteoclasts to induce bone resorption. These results are further supported by recent findings, which demonstrated the presence of a specific pro-inflammatory transcriptional phenotype in MM-associated MSC, which could persist even after deep responses and in patients with a MRD+ disease [76,77]. It is, therefore, clear that targeting this interplay trough RANK network disrupters (denosumab) or with proteasome inhibitors such as ixazomib, that have been demonstrated to induce an osteogenic differentiation from MSC, could be of the utmost importance to counteract the pro-tumor effects of the MSC-dependent inflammatory microenvironment [78].

## 5. Long-Term Failure of Novel Immunotherapies: Pitfalls and Opportunities

Despite the exceptional response rates observed with several new immunotherapies (BCMA-targeted CAR T cells and bispecific T cells engagers), a significant proportion of patients systematically relapse [79]. Unfortunately, the mechanisms underlying therapy failures are far from being elucidated. Among others, antigen downregulation, the dependence on T-cell “fitness” as well as the presence of an immunosuppressive BM-IM could potentially play an important role in the establishment of a long-term resistance to immune-based therapies in these patients. Specifically, it has been observed that for BCMA-targeting agents, antigen deregulation could happen as a consequence of previous anti-BCMA treatments (e.g., belantamab) or by genetic alterations. Indeed, biallelic loss of BCMA has been reported in several patients relapsing after anti-BCMA CAR-T [80,81]. Additionally, after a deeper analysis, a heterozygous TNFRSF17 loss or monosomy of 16 in about 15% of MM patients never exposed to anti-BCMA therapy has been observed, letting us hypothesize a role for genomic alteration in the development of resistance to BCMA-targeting treatments [80,81]. On the other hand, the activity of anti-CD3/BCMA bispecific antibodies have been found to be completely abrogated by the presence of granulocyte-like MDSC [16] or dysfunctional/exhausted T-cells, which arise under an immunosuppressive microenvironment characterized by a repeated T-cell stimulation [79]. Interestingly, while the targeting of the PD1/PD-L1 axis in MM patients never demonstrated a significant clinical benefit [82,83,84], the possibility to combine these drugs with bispecifics or cell therapy is currently under study due to the overexpression of PD1 on exhausted (CAR-)T cells. Preliminary clinical evidence suggested, indeed, the possibility to prolong the persistence of adoptive T cells once these were genetically engineered with a disrupted PDCD1 gene [85]. A further promising approach relies on the combination of immunomodulatory agents (such as lenalidomide) with CAR-T. Along this line, preclinical studies demonstrated that by enhancing the production of Th1 cytokines, lenalidomide increases CAR-T activity even within immunosuppressive environments and delays the onset of T cell exhaustion [79,86]. New combinatory strategies, which aim to overcome the resistance induced by the BM-IM (including the use of novel CELMoDs), are currently under active preclinical and clinical investigation, and results are eagerly awaited [79,87].

## 6. Conclusions

The evolution from MGUS to active MM is associated with a complex and extensive reprogramming of the whole immune contexture. Unfortunately, most of these changes are still far to be elucidated entirely. Many efforts should be made to identify new biomarkers and novel therapeutic targets to prevent disease evolution. Indeed, much evidence from solid tumors underlines how the road to cure cancer includes treating patients early. Along this line, several trials on early treatment of asymptomatic (but at high risk of progression) MM with IMiDs or mAbs showed advantages in terms of both time to disease evolution and survival.

Additionally, it is of extreme relevance the identification of potential immunomodulatory activities of drugs currently used for the treatment of advanced disease (such as proteasome inhibitors [88]) and of novel molecules (hypomethylating agents, HDAC inhibitors, checkpoint inhibitors) [16,49,60,62]. This will lead to the translational design of innovative combination strategies tailored to each patient’s “immune status” and to the development of personalized immune therapy for symptomatic (or asymptomatic) MM disease.

## References

[1] Cusi M.G., Botta C., Pastina P., Rossetti M.G., Dreassi E., Guidelli G.M., Fioravanti A., Martino E.C., Gandolfo C., Pagliuchi M. (2015). Phase I trial of thymidylate synthase poly-epitope peptide (TSPP) vaccine in advanced cancer patients. Cancer Immunol. Immunother..

[2] Botta C., Misso G., Martino E.C., Pirtoli L., Cusi M.G., Tassone P., Tagliaferri P., Caraglia M., Correale P. (2016). The route to solve the interplay between inflammation, angiogenesis and anti-cancer immune response. Cell Death Dis..

[3] Botta C., Bestoso E., Apollinari S., Cusi M.G., Pastina P., Abbruzzese A., Sperlongano P., Misso G., Caraglia M., Tassone P. (2012). Immune-modulating effects of the newest cetuximab-based chemoimmunotherapy regimen in advanced colorectal cancer patients. J. Immunother..

[4] Nakamura K., Smyth M.J., Martinet L. (2020). Cancer immunoediting and immune dysregulation in multiple myeloma. Blood.

[5] Gupta R.G., Li F., Roszik J., Lizee G. (2021). Exploiting Tumor Neoantigens to Target Cancer Evolution: Current Challenges and Promising Therapeutic Approaches. Cancer Discov..

[6] Kakiuchi N., Ogawa S. (2021). Clonal expansion in non-cancer tissues. Nat. Rev. Cancer.

[7] Botta C., Di Martino M.T., Ciliberto D., Cuce M., Correale P., Rossi M., Tagliaferri P., Tassone P. (2016). A gene expression inflammatory signature specifically predicts multiple myeloma evolution and patients survival. Blood Cancer J..

[8] Lomas O.C., Mouhieddine T.H., Tahri S., Ghobrial I.M. (2020). Monoclonal Gammopathy of Undetermined Significance (MGUS)-Not So Asymptomatic after All. Cancers.

[9] Bustoros M., Sklavenitis-Pistofidis R., Park J., Redd R., Zhitomirsky B., Dunford A.J., Salem K., Tai Y.T., Anand S., Mouhieddine T.H. (2020). Genomic Profiling of Smoldering Multiple Myeloma Identifies Patients at a High Risk of Disease Progression. J. Clin. Oncol..

[10] Botta C., Martino E.A., Conticello C., Mendicino F., Vigna E., Romano A., Palumbo G.A., Cerchione C., Martinelli G., Morabito F. (2021). Treatment of lenalidomide exposed or refractory multiple myeloma: Network meta-analysis of lenalidomide-sparing regimens. Front. Oncol..

[11] Botta C., Ciliberto D., Rossi M., Staropoli N., Cuce M., Galeano T., Tagliaferri P., Tassone P. (2017). Network meta-analysis of randomized trials in multiple myeloma: Efficacy and safety in relapsed/refractory patients. Blood Adv..

[12] Leone E., Morelli E., Di Martino M.T., Amodio N., Foresta U., Gulla A., Rossi M., Neri A., Giordano A., Munshi N.C. (2013). Targeting miR-21 inhibits in vitro and in vivo multiple myeloma cell growth. Clin. Cancer Res..

[13] Pitari M.R., Rossi M., Amodio N., Botta C., Morelli E., Federico C., Gulla A., Caracciolo D., Di Martino M.T., Arbitrio M. (2015). Inhibition of miR-21 restores RANKL/OPG ratio in multiple myeloma-derived bone marrow stromal cells and impairs the resorbing activity of mature osteoclasts. Oncotarget.

[14] Botta C., Cuce M., Pitari M.R., Caracciolo D., Gulla A., Morelli E., Riillo C., Biamonte L., Gallo Cantafio M.E., Prabhala R. (2018). MiR-29b antagonizes the pro-inflammatory tumor-promoting activity of multiple myeloma-educated dendritic cells. Leukemia.

[15] Botta C., Gulla A., Correale P., Tagliaferri P., Tassone P. (2014). Myeloid-derived suppressor cells in multiple myeloma: Pre-clinical research and translational opportunities. Front. Oncol..

[16] Perez C., Botta C., Zabaleta A., Puig N., Cedena M.T., Goicoechea I., Alameda D., San Jose-Eneriz E., Merino J., Rodriguez-Otero P. (2020). Immunogenomic identification and characterization of granulocytic myeloid-derived suppressor cells in multiple myeloma. Blood.

[17] Pazina T., MacFarlane A.W.t., Bernabei L., Dulaimi E., Kotcher R., Yam C., Bezman N.A., Robbins M.D., Ross E.A., Campbell K.S. (2021). Alterations of NK Cell Phenotype in the Disease Course of Multiple Myeloma. Cancers.

[18] Rossi M., Altomare E., Botta C., Gallo Cantafio M.E., Sarvide S., Caracciolo D., Riillo C., Gaspari M., Taverna D., Conforti F. (2021). miR-21 antagonism abrogates Th17 tumor promoting functions in multiple myeloma. Leukemia.

[19] Prabhala R.H., Fulciniti M., Pelluru D., Rashid N., Nigroiu A., Nanjappa P., Pai C., Lee S., Prabhala N.S., Bandi R.L. (2016). Targeting IL-17A in multiple myeloma: A potential novel therapeutic approach in myeloma. Leukemia.

[20] Calcinotto A., Brevi A., Chesi M., Ferrarese R., Garcia Perez L., Grioni M., Kumar S., Garbitt V.M., Sharik M.E., Henderson K.J. (2018). Microbiota-driven interleukin-17-producing cells and eosinophils synergize to accelerate multiple myeloma progression. Nat. Commun..

[21] Ahmed N., Ghannoum M., Gallogly M., de Lima M., Malek E. (2020). Influence of gut microbiome on multiple myeloma: Friend or foe?. J. Immunother. Cancer.

[22] Botta C., Cuce M., Caracciolo D., Fiorillo L., Tagliaferri P., Tassone P. (2017). Immunomodulatory Activity of MicroRNAs: Potential Implications for Multiple Myeloma Treatment. Curr. Cancer Drug Targets.

[23] Leone P., Solimando A.G., Malerba E., Fasano R., Buonavoglia A., Pappagallo F., De Re V., Argentiero A., Silvestris N., Vacca A. (2020). Actors on the Scene: Immune Cells in the Myeloma Niche. Front. Oncol..

[24] Rossi M., Botta C., Correale P., Tassone P., Tagliaferri P. (2013). Immunologic microenvironment and personalized treatment in multiple myeloma. Expert Opin. Biol. Ther..

[25] Díaz-Tejedor A., Lorenzo-Mohamed M., Puig N., García-Sanz R., Mateos M.-V., Garayoa M., Paíno T. (2021). Immune System Alterations in Multiple Myeloma: Molecular Mechanisms and Therapeutic Strategies to Reverse Immunosuppression. Cancers.

[26] Gorgun G.T., Whitehill G., Anderson J.L., Hideshima T., Maguire C., Laubach J., Raje N., Munshi N.C., Richardson P.G., Anderson K.C. (2013). Tumor-promoting immune-suppressive myeloid-derived suppressor cells in the multiple myeloma microenvironment in humans. Blood.

[27] Ramachandran I.R., Martner A., Pisklakova A., Condamine T., Chase T., Vogl T., Roth J., Gabrilovich D., Nefedova Y. (2013). Myeloid-derived suppressor cells regulate growth of multiple myeloma by inhibiting T cells in bone marrow. J. Immunol..

[28] Lopes R., Caetano J., Ferreira B., Barahona F., Carneiro E.A., Joao C. (2021). The Immune Microenvironment in Multiple Myeloma: Friend or Foe?. Cancers.

[29] Ramachandran I.R., Condamine T., Lin C., Herlihy S.E., Garfall A., Vogl D.T., Gabrilovich D.I., Nefedova Y. (2016). Bone marrow PMN-MDSCs and neutrophils are functionally similar in protection of multiple myeloma from chemotherapy. Cancer Lett..

[30] Puglisi F., Parrinello N.L., Giallongo C., Cambria D., Camiolo G., Bellofiore C., Conticello C., Del Fabro V., Leotta V., Markovic U. (2019). Plasticity of High-Density Neutrophils in Multiple Myeloma is Associated with Increased Autophagy via STAT3. Int. J. Mol. Sci..

[31] Romano A., Parrinello N.L., Simeon V., Puglisi F., La Cava P., Bellofiore C., Giallongo C., Camiolo G., D’Auria F., Grieco V. (2020). High-density neutrophils in MGUS and multiple myeloma are dysfunctional and immune-suppressive due to increased STAT3 downstream signaling. Sci. Rep..

[32] Romano A., Laura Parrinello N., Cerchione C., Letizia Consoli M., Parisi M., Calafiore V., Martino E., Conticello C., Di Raimondo F., Alberto Palumbo G. (2017). The NLR and LMR ratio in newly diagnosed MM patients treated upfront with novel agents. Blood Cancer J..

[33] Solmaz Medeni S., Acar C., Olgun A., Acar A., Seyhanli A., Taskiran E., Sevindik O.G., Alacacioglu I., Piskin O., Ozcan M.A. (2018). Can Neutrophil-to-Lymphocyte Ratio, Monocyte-to-Lymphocyte Ratio, and Platelet-to-Lymphocyte Ratio at Day +100 be used as a prognostic marker in Multiple Myeloma patients with autologous transplantation?. Clin. Transplant..

[34] Beyar-Katz O., Magidey K., Reiner-Benaim A., Barak N., Avivi I., Cohen Y., Timaner M., Avraham S., Hayun M., Lavi N. (2019). Proinflammatory Macrophages Promote Multiple Myeloma Resistance to Bortezomib Therapy. Mol. Cancer Res..

[35] Zheng Y., Cai Z., Wang S., Zhang X., Qian J., Hong S., Li H., Wang M., Yang J., Yi Q. (2009). Macrophages are an abundant component of myeloma microenvironment and protect myeloma cells from chemotherapy drug-induced apoptosis. Blood.

[36] Sacco A., Battaglia A.M., Botta C., Aversa I., Mancuso S., Costanzo F., Biamonte F. (2021). Iron Metabolism in the Tumor Microenvironment-Implications for Anti-Cancer Immune Response. Cells.

[37] Oshi M., Tokumaru Y., Asaoka M., Yan L., Satyananda V., Matsuyama R., Matsuhashi N., Futamura M., Ishikawa T., Yoshida K. (2020). M1 Macrophage and M1/M2 ratio defined by transcriptomic signatures resemble only part of their conventional clinical characteristics in breast cancer. Sci. Rep..

[38] Wu K., Lin K., Li X., Yuan X., Xu P., Ni P., Xu D. (2020). Redefining Tumor-Associated Macrophage Subpopulations and Functions in the Tumor Microenvironment. Front. Immunol..

[39] Damasceno D., Almeida J., Teodosio C., Sanoja-Flores L., Mayado A., Perez-Pons A., Puig N., Arana P., Paiva B., Solano F. (2021). Monocyte Subsets and Serum Inflammatory and Bone-Associated Markers in Monoclonal Gammopathy of Undetermined Significance and Multiple Myeloma. Cancers.

[40] Andersen M.N., Andersen N.F., Rodgaard-Hansen S., Hokland M., Abildgaard N., Moller H.J. (2015). The novel biomarker of alternative macrophage activation, soluble mannose receptor (sMR/sCD206): Implications in multiple myeloma. Leuk. Res..

[41] Panchabhai S., Kelemen K., Ahmann G., Sebastian S., Mantei J., Fonseca R. (2016). Tumor-associated macrophages and extracellular matrix metalloproteinase inducer in prognosis of multiple myeloma. Leukemia.

[42] Suyani E., Sucak G.T., Akyurek N., Sahin S., Baysal N.A., Yagci M., Haznedar R. (2013). Tumor-associated macrophages as a prognostic parameter in multiple myeloma. Ann. Hematol..

[43] Zavidij O., Haradhvala N.J., Mouhieddine T.H., Sklavenitis-Pistofidis R., Cai S., Reidy M., Rahmat M., Flaifel A., Ferland B., Su N.K. (2020). Single-cell RNA sequencing reveals compromised immune microenvironment in precursor stages of multiple myeloma. Nat. Cancer.

[44] Mougiakakos D., Bach C., Bottcher M., Beier F., Rohner L., Stoll A., Rehli M., Gebhard C., Lischer C., Eberhardt M. (2021). The IKZF1-IRF4/IRF5 Axis Controls Polarization of Myeloma-Associated Macrophages. Cancer Immunol. Res..

[45] Moreno L., Perez C., Zabaleta A., Manrique I., Alignani D., Ajona D., Blanco L., Lasa M., Maiso P., Rodriguez I. (2019). The Mechanism of Action of the Anti-CD38 Monoclonal Antibody Isatuximab in Multiple Myeloma. Clin. Cancer Res..

[46] Naicker S.D., Feerick C.L., Lynch K., Swan D., McEllistrim C., Henderson R., Leonard N.A., Treacy O., Natoni A., Rigalou A. (2021). Cyclophosphamide alters the tumor cell secretome to potentiate the anti-myeloma activity of daratumumab through augmentation of macrophage-mediated antibody dependent cellular phagocytosis. Oncoimmunology.

[47] Sun J., Muz B., Alhallak K., Markovic M., Gurley S., Wang Z., Guenthner N., Wasden K., Fiala M., King J. (2020). Targeting CD47 as a Novel Immunotherapy for Multiple Myeloma. Cancers.

[48] Camiolo G., Barbato A., Giallongo C., Vicario N., Romano A., Parrinello N.L., Parenti R., Sandoval J.C., Garcia-Moreno D., Lazzarino G. (2020). Iron regulates myeloma cell/macrophage interaction and drives resistance to bortezomib. Redox. Biol..

[49] Cuce M., Gallo Cantafio M.E., Siciliano M.A., Riillo C., Caracciolo D., Scionti F., Staropoli N., Zuccala V., Maltese L., Di Vito A. (2019). Trabectedin triggers direct and NK-mediated cytotoxicity in multiple myeloma. J. Hematol. Oncol..

[50] Correale P., Botta C., Cusi M.G., Del Vecchio M.T., De Santi M.M., Gori Savellini G., Bestoso E., Apollinari S., Mannucci S., Marra M. (2012). Cetuximab +/- chemotherapy enhances dendritic cell-mediated phagocytosis of colon cancer cells and ignites a highly efficient colon cancer antigen-specific cytotoxic T-cell response in vitro. Int. J. Cancer.

[51] Leone P., Berardi S., Frassanito M.A., Ria R., De Re V., Cicco S., Battaglia S., Ditonno P., Dammacco F., Vacca A. (2015). Dendritic cells accumulate in the bone marrow of myeloma patients where they protect tumor plasma cells from CD8+ T-cell killing. Blood.

[52] Koduru S., Wong E., Strowig T., Sundaram R., Zhang L., Strout M.P., Flavell R.A., Schatz D.G., Dhodapkar K.M., Dhodapkar M.V. (2012). Dendritic cell-mediated activation-induced cytidine deaminase (AID)-dependent induction of genomic instability in human myeloma. Blood.

[53] Chung D.J., Rossi M., Romano E., Ghith J., Yuan J., Munn D.H., Young J.W. (2009). Indoleamine 2,3-dioxygenase-expressing mature human monocyte-derived dendritic cells expand potent autologous regulatory T cells. Blood.

[54] Murray M.E., Gavile C.M., Nair J.R., Koorella C., Carlson L.M., Buac D., Utley A., Chesi M., Bergsagel P.L., Boise L.H. (2014). CD28-mediated pro-survival signaling induces chemotherapeutic resistance in multiple myeloma. Blood.

[55] Bahlis N.J., King A.M., Kolonias D., Carlson L.M., Liu H.Y., Hussein M.A., Terebelo H.R., Byrne G.E., Levine B.L., Boise L.H. (2007). CD28-mediated regulation of multiple myeloma cell proliferation and survival. Blood.

[56] Phan V., Ito T., Inaba M., Azuma Y., Kibata K., Inagaki-Katashiba N., Tanaka A., Satake A., Nomura S. (2020). Immunomodulatory drugs suppress Th1-inducing ability of dendritic cells but enhance Th2-mediated allergic responses. Blood Adv..

[57] Foglietta M., Castella B., Mariani S., Coscia M., Godio L., Ferracini R., Ruggeri M., Muccio V., Omede P., Palumbo A. (2014). The bone marrow of myeloma patients is steadily inhabited by a normal-sized pool of functional regulatory T cells irrespectiveof the disease status. Haematologica.

[58] Atanackovic D., Cao Y., Luetkens T., Panse J., Faltz C., Arfsten J., Bartels K., Wolschke C., Eiermann T., Zander A.R. (2008). CD4+CD25+FOXP3+ T regulatory cells reconstitute and accumulate in the bone marrow of patients with multiple myeloma following allogeneic stem cell transplantation. Haematologica.

[59] Feng X., Zhang L., Acharya C., An G., Wen K., Qiu L., Munshi N.C., Tai Y.T., Anderson K.C. (2017). Targeting CD38 Suppresses Induction and Function of T Regulatory Cells to Mitigate Immunosuppression in Multiple Myeloma. Clin. Cancer Res..

[60] Minnie S.A., Kuns R.D., Gartlan K.H., Zhang P., Wilkinson A.N., Samson L., Guillerey C., Engwerda C., MacDonald K.P.A., Smyth M.J. (2018). Myeloma escape after stem cell transplantation is a consequence of T-cell exhaustion and is prevented by TIGIT blockade. Blood.

[61] Quarona V., Ferri V., Chillemi A., Bolzoni M., Mancini C., Zaccarello G., Roato I., Morandi F., Marimpietri D., Faccani G. (2015). Unraveling the contribution of ectoenzymes to myeloma life and survival in the bone marrow niche. Ann. N. Y. Acad. Sci..

[62] Hradska K., Kascak M., Hajek R., Jelinek T. (2020). Identifying and treating candidates for checkpoint inhibitor therapies in multiple myeloma and lymphoma. Expert Rev. Hematol..

[63] Oriol A. (2020). A critical evaluation of pembrolizumab in addition to lenalidomide and dexamethasone for the treatment of multiple myeloma. Expert Rev. Hematol..

[64] Minnie S.A., Hill G.R. (2020). Immunotherapy of multiple myeloma. J. Clin. Investig..

[65] Lozano E., Mena M.P., Diaz T., Martin-Antonio B., Leon S., Rodriguez-Lobato L.G., Oliver-Caldes A., Cibeira M.T., Blade J., Prat A. (2020). Nectin-2 Expression on Malignant Plasma Cells Is Associated with Better Response to TIGIT Blockade in Multiple Myeloma. Clin. Cancer Res..

[66] Leivas A., Risueno R.M., Guzman A., Sanchez-Vega L., Perez M., Megias D., Fernandez L., Alonso R., Perez-Martinez A., Rapado I. (2021). Natural killer cells efficiently target multiple myeloma clonogenic tumor cells. Cancer Immunol. Immunother..

[67] Fionda C., Stabile H., Molfetta R., Soriani A., Bernardini G., Zingoni A., Gismondi A., Paolini R., Cippitelli M., Santoni A. (2018). Translating the anti-myeloma activity of Natural Killer cells into clinical application. Cancer Treat. Rev..

[68] Soriani A., Iannitto M.L., Ricci B., Fionda C., Malgarini G., Morrone S., Peruzzi G., Ricciardi M.R., Petrucci M.T., Cippitelli M. (2014). Reactive oxygen species- and DNA damage response-dependent NK cell activating ligand upregulation occurs at transcriptional levels and requires the transcriptional factor E2F1. J. Immunol..

[69] Soriani A., Fionda C., Ricci B., Iannitto M.L., Cippitelli M., Santoni A. (2013). Chemotherapy-elicited upregulation of NKG2D and DNAM-1 ligands as a therapeutic target in multiple myeloma. Oncoimmunology.

[70] Gentile M., Specchia G., Derudas D., Galli M., Botta C., Rocco S., Conticello C., Califano C., Giuliani N., Mangiacavalli S. (2021). Elotuzumab, lenalidomide, and dexamethasone as salvage therapy for patients with multiple myeloma: Italian, multicenter, retrospective clinical experience with 300 cases outside of controlled clinical trials. Haematologica.

[71] Radocha J., van de Donk N., Weisel K. (2021). Monoclonal Antibodies and Antibody Drug Conjugates in Multiple Myeloma. Cancers.

[72] Sarvaria A., Madrigal J.A., Saudemont A. (2017). B cell regulation in cancer and anti-tumor immunity. Cell Mol. Immunol..

[73] Zhang L., Tai Y.T., Ho M., Xing L., Chauhan D., Gang A., Qiu L., Anderson K.C. (2017). Regulatory B cell-myeloma cell interaction confers immunosuppression and promotes their survival in the bone marrow milieu. Blood Cancer J..

[74] Krejcik J., Casneuf T., Nijhof I.S., Verbist B., Bald J., Plesner T., Syed K., Liu K., van de Donk N.W., Weiss B.M. (2016). Daratumumab depletes CD38+ immune regulatory cells, promotes T-cell expansion, and skews T-cell repertoire in multiple myeloma. Blood.

[75] Zou Z., Guo T., Cui J., Tang W., Li Y., Wang F., Dong T., Yang Y., Feng Y., Ho M. (2021). Real-world data combined with studies on Regulatory B Cells for newly diagnosed Multiple Myeloma from a tertiary referral Hospital in South-Western China. J. Cancer.

[76] Alameda D., Saez B., Lara-Astiaso D., Sarvide S., Lasa M., Alignani D., Rodriguez I., Garate S., Vilas A., Paiva B. (2020). Characterization of freshly isolated bone marrow mesenchymal stromal cells from healthy donors and patients with multiple myeloma: Transcriptional modulation of the microenvironment. Haematologica.

[77] de Jong M.M.E., Kellermayer Z., Papazian N., Tahri S., Hofste Op Bruinink D., Hoogenboezem R., Sanders M.A., van de Woestijne P.C., Bos P.K., Khandanpour C. (2021). The multiple myeloma microenvironment is defined by an inflammatory stromal cell landscape. Nat Immunol..

[78] Tibullo D., Longo A., Vicario N., Romano A., Barbato A., Di Rosa M., Barbagallo I., Anfuso C.D., Lupo G., Gulino R. (2020). Ixazomib Improves Bone Remodeling and Counteracts sonic Hedgehog signaling Inhibition Mediated by Myeloma Cells. Cancers.

[79] Wudhikarn K., Mailankody S., Smith E.L. (2020). Future of CAR T cells in multiple myeloma. Hematology Am. Soc. Hematol. Educ. Program..

[80] Samur M.K., Fulciniti M., Aktas Samur A., Bazarbachi A.H., Tai Y.T., Prabhala R., Alonso A., Sperling A.S., Campbell T., Petrocca F. (2021). Biallelic loss of BCMA as a resistance mechanism to CAR T cell therapy in a patient with multiple myeloma. Nat. Commun..

[81] Da Via M.C., Dietrich O., Truger M., Arampatzi P., Duell J., Heidemeier A., Zhou X., Danhof S., Kraus S., Chatterjee M. (2021). Homozygous BCMA gene deletion in response to anti-BCMA CAR T cells in a patient with multiple myeloma. Nat. Med..

[82] Frerichs K.A., Verkleij C.P.M., Dimopoulos M.A., Marin Soto J.A., Zweegman S., Young M.H., Newhall K.J., Mutis T., van de Donk N. (2021). Efficacy and Safety of Durvalumab Combined with Daratumumab in Daratumumab-Refractory Multiple Myeloma Patients. Cancers.

[83] Usmani S.Z., Schjesvold F., Oriol A., Karlin L., Cavo M., Rifkin R.M., Yimer H.A., LeBlanc R., Takezako N., McCroskey R.D. (2019). Pembrolizumab plus lenalidomide and dexamethasone for patients with treatment-naive multiple myeloma (KEYNOTE-185): A randomised, open-label, phase 3 trial. Lancet Haematol..

[84] Puig N., Corchete-Sanchez L.A., Perez-Moran J.J., Davila J., Paino T., de la Rubia J., Oriol A., Martin-Sanchez J., de Arriba F., Blade J. (2020). Pembrolizumab as Consolidation Strategy in Patients with Multiple Myeloma: Results of the GEM-Pembresid Clinical Trial. Cancers.

[85] Stadtmauer E.A., Fraietta J.A., Davis M.M., Cohen A.D., Weber K.L., Lancaster E., Mangan P.A., Kulikovskaya I., Gupta M., Chen F. (2020). CRISPR-engineered T cells in patients with refractory cancer. Science.

[86] Works M., Soni N., Hauskins C., Sierra C., Baturevych A., Jones J.C., Curtis W., Carlson P., Johnstone T.G., Kugler D. (2019). Anti-B-cell Maturation Antigen Chimeric Antigen Receptor T cell Function against Multiple Myeloma Is Enhanced in the Presence of Lenalidomide. Mol. Cancer Ther..

[87] Maples K.T., Joseph N.S., Harvey R.D. (2020). Current developments in the combination therapy of relapsed/refractory multiple myeloma. Expert Rev. Anticancer Ther..

[88] Gulla A., Morelli E., Samur M.K., Botta C., Hideshima T., Bianchi G., Fulciniti M., Malvestiti S., Prabhala R.H., Talluri S. (2021). Bortezomib induces anti-multiple myeloma immune response mediated by cGAS/STING pathway activation. Blood Cancer Discov..

